# Identification of miRNA-mRNA Regulatory Networks Reveals Potential Key miRNAs in Goat Intramuscular and Subcutaneous Adipose Formation

**DOI:** 10.3390/ani16162603

**Published:** 2026-08-20

**Authors:** Jiani Xing, Huifeng Ma, Jianmei Wang, Yaqiu Lin, Yong Wang, Fulai Xue, Xin Li, Yaohui Dong, Yanyan Li, Youli Wang, Haitao Shi, Wei Liu

**Affiliations:** 1 Key Laboratory of Qinghai-Tibetan Plateau Animal Genetic Resource Reservation and Utilization, Ministry of Education, Southwest Minzu University, Chengdu 610041, China; 80300244@swun.edu.cn (J.X.); 18896140051@163.com (H.M.); linyq1999@163.com (Y.L.);; 2 Key Laboratory of Qinghai-Tibetan Plateau Animal Genetic Resource Reservation and Exploitation of Sichuan Province, Southwest Minzu University, Chengdu 610041, China; 3 College of Animal & Veterinary Science, Southwest Minzu University, Chengdu 610041, China

**Keywords:** miRNA, mRNA, intramuscular fat, subcutaneous fat, goat

## Abstract

The differential fat deposition in intramuscular (IMF) and subcutaneous (SCF) adipose tissues significantly influences goat meat quality. While microRNAs (miRNAs) are key post-transcriptional regulators, the shared key miRNAs governing adipogenesis across different adipose depots remain largely unexplored in goats. In this study, intramuscular and subcutaneous preadipocytes were isolated from the same healthy 7-day-old male Jianzhou Da’er goat, and each experimental group comprised five independently cultured cell replicates derived from this donor. Integrated miRNA and mRNA sequencing was performed on undifferentiated intramuscular (JNE) and subcutaneous (PE) preadipocytes and their corresponding adipocytes after 72 h of differentiation (JNC and PC, respectively), allowing the identification of depot-specific and shared differentially expressed miRNAs (DEmiRNAs) and mRNAs (DEmRNAs). By constructing miRNA–mRNA regulatory networks, chi-miR-1 was identified as the highest-degree miRNA node in the shared network and was therefore prioritized for functional evaluation. RT-qPCR, chi-miR-1 mimic and inhibitor transfection, Oil Red O staining, and BODIPY staining showed that chi-miR-1 overexpression increased lipid droplet accumulation, whereas its inhibition produced the opposite effect in both intramuscular and subcutaneous adipocytes. Furthermore, bioinformatic target prediction and network analysis identified chi-miR-1–PPARG and chi-miR-206–IGFBP3 as predicted potential regulatory relationships; these interactions were not directly validated experimentally. Our findings, obtained using cells derived from a single donor, suggest that chi-miR-1 may serve as a candidate shared pro-adipogenic regulator under the present in vitro conditions and provide insights into miRNA-mediated regulation of adipogenesis in different goat adipose depots.

## Simple Summary

Fat deposition within muscle and beneath the skin influences the flavor, texture, and commercial value of goat meat. However, the molecular mechanisms controlling fat cell formation in these two locations are not fully understood. In this study, we compared changes in microRNAs and their potential target genes before and after fat cell formation in cells derived from goat muscle and subcutaneous tissues. By combining gene expression analysis with computational prediction of regulatory relationships, we identified both depot-specific and shared regulatory patterns. Among the shared microRNAs, chi-miR-1 showed the greatest number of predicted regulatory connections and was therefore selected for further experimental validation. Increasing chi-miR-1 expression promoted lipid droplet accumulation in both types of fat cells, whereas reducing its expression resulted in the opposite effect. These findings suggest that chi-miR-1 may participate in a shared regulatory process controlling fat cell formation in different goat adipose depots. This study improves our understanding of fat deposition in different goat tissues and provides valuable information for further investigation of the biological basis regulating goat meat quality.

## 1. Introduction

Adipose tissues are located in different parts of the body, and in domestic animals, they are mainly divided into subcutaneous adipose tissue (SCF) and intramuscular adipose tissue (IMF). Fat deposition varies among breeds and can be influenced by many factors, among which genetic factors are considered major determinants.

Goat meat is one of the major sources of animal meat production for human consumption. In goat breeding, the IMF content is an important consideration and a determining factor, as it is highly related to meat quality and consumer palatability [1]. Prolonging the fattening period and providing a high-energy diet can increase the IMF content but can also lead to excessive external fat deposition, such as subcutaneous and perirenal fat accumulation [2,3]. It has been shown that there are differences in the physiological functions and lipid metabolism of intramuscular, subcutaneous, and perirenal adipocytes due to their location [4,5,6]. Previous studies have shown that adipocytes from different anatomical depots differ in their physiological functions and lipid metabolism [7,8]. Therefore, elucidating the molecular mechanisms underlying fat deposition in different adipose tissues is important for understanding goat adipose development and improving meat quality.

Adipogenesis is a complicated process regulated by various transcription factors, key genes, and non-coding RNAs. Recently, more and more studies have indicated that non-coding RNAs, including microRNAs (miRNAs), long non-coding RNAs (lncRNAs), and circular RNAs (circRNAs), play pivotal roles in various biological processes [9]. MiRNAs are endogenous non-coding RNAs approximately 22 nucleotides in length that bind complementary target mRNAs and regulate gene expression post-transcriptionally [10]. With the development of high-throughput sequencing technologies, emerging evidence has suggested the significant role of miRNAs in regulating the development of adipose tissue and the differentiation process of adipocytes. For instance, miR-378 has a strong correlation with backfat thickness in beef cattle [11]. Using RNA sequencing, Huang et al. [12] analyzed the expression of mRNA and miRNA in the livers of Jinhua (JH) pigs and Landrace (LD) pigs and identified 35 miRNAs and 467 genes associated with fat metabolism and deposition, respectively. Other miRNAs, including miR-27b [13], miR-103 [14], and miR-148a [15], were closely associated with the regulation of adipogenesis in animals, either promoting or inhibiting adipogenesis. Nevertheless, compared to other livestock species, the molecular mechanisms of miRNA–mRNA interactions in goat adipogenesis remain poorly understood.

Therefore, we hypothesized that the differentiation process of goat intramuscular and subcutaneous preadipocytes is regulated by both depot-specific and shared miRNA–mRNA regulatory mechanisms and that shared hub miRNAs may play crucial roles in adipogenesis across these two adipose depots. To test this hypothesis, we first performed integrated miRNA and mRNA expression profiling to identify differentially expressed and shared miRNAs and mRNAs during the differentiation of goat intramuscular and subcutaneous preadipocytes. We then constructed depot-specific and shared miRNA–mRNA regulatory networks to identify potential key regulatory miRNAs and their predicted target relationships. Finally, chi-miR-1, a candidate hub miRNA shared by both adipocyte types, was selected for functional evaluation using RT-qPCR, miRNA mimic and inhibitor transfection, Oil Red O staining, and BODIPY staining. The results of this study provided novel insights into miRNAs and genes with respect to the regulatory mechanisms underlying adipocyte differentiation and fat deposition in different anatomical fat depots in goat meat.

## 2. Materials and Methods

### 2.1. Isolation and Culture of Goat Subcutaneous and Intramuscular Preadipocytes

All experimental procedures were reviewed and approved by the Institutional Animal Care and Use Committee, Southwest Minzu University (Chengdu, China). Under sterile conditions, the abdominal subcutaneous and intramuscular fat tissues were separated from a healthy 7-day-old male Jianzhou Da’er goat by removing the connective tissue and blood vessels. After washing 2~3 times with PBS, an equal volume of culture medium (DMEM/F12 + 15% FBS + 2% penicillin–streptomycin solution, Gibco, Grand Island, NY, USA) was added to terminate the digestion after 90 min of digestion using Type II collagenase (Sigma-Aldrich, St. Louis, MO, USA), and the medium was filtered through a 400-mesh filter and centrifuged at 2000 r/min for 5 min. A red blood cell lysate (biosharp, BL503A) was added to the pellet to lyse for 5 min, and then, the supernatant was removed by centrifugation. An appropriate amount of culture medium was added to suspend the cell pellet, and the cells were seeded in a 25 cm^2^ culture flask to obtain primary intramuscular and subcutaneous adipocytes.

### 2.2. Cell Culture and Adipogenic Differentiation Induction of Goat Intramuscular and Subcutaneous Adipocytes

When the primary intramuscular and subcutaneous adipocytes reached 70–80% confluence, the cells were transferred to the F2 generation, the medium was discarded, and the cells were rinsed slowly twice with PBS and then digested with 1 mL of trypsin at room temperature for 1 min. After digestion, 6 mL of culture medium was added to stop the digestion and resuspend the cells, and then, the cells were transferred into 10 mL centrifugal tubes and centrifuged at 2000 r/min for 2 min. The supernatant was discarded, and the culture medium was added for resuspension. The cells were then inoculated into 10 mm dishes and were observed every 24 h with changing the medium. When the cells fused reached 70–80% confluence—i.e., the F3 generation (intramuscular adipocytes were recorded as the JNE group, and subcutaneous adipocytes were recorded as the PE group)—cell differentiation was induced using 50 μmol/L oleic acid, and the cells were harvested after 72 h of differentiation (intramuscular adipocytes were recorded as the JNC group, and subcutaneous adipocytes were recorded as the PC group), with five replicates per group.

### 2.3. Oil Red O Staining, BODIPY Staining, and DAPI Staining

After 2-day adipogenic differentiation induction, the medium was replaced with growth medium for one day, and the cells assigned to the PC group and JNC group were collected. After washing twice with PBS, the cells were fixed with 4% paraformaldehyde for 30 min and washed with PBS and then stained with Oil Red O solution for 30 min. After washing with PBS three times, the size of intracellular lipid droplets was observed, and pictures were taken under the microscope. Subsequently, isopropanol was added to dissolve the Oil Red O, and the absorbance value was measured at 490 nm.

BODIPY working solutions (1:1000 dilution in PBS) were added to each well at 200 μL per well and incubated in the dark for 15 min. Then, DAPI dye (diluted similarly) was added for another 10 min of incubation. After two washes with PBS, 200 μL of PBS was added, and lipid droplet morphology was examined and imaged using a fluorescence microscope (Olympus, Tokyo, Japan).

The preadipocytes were assigned to the PE group and JNE group. The detailed grouping, differentiation status, sampling time points, and replicate structure were summarized in Table 1.

### 2.4. Library Construction and RNA Sequencing

The total RNA was extracted using TRIzol (Takara, Bio Inc., Kusatsu, Shiga, Japan) and was verified on 1% agarose gels. RNA purity was checked using the Nano-Photometer^®^ spectrophotometer (Implen, Inc., Westlake Village, CA, USA), and the concentration was measured using a Qubit^®^ RNA Assay Kit with a Qubit^®^ 2.0 Flurometer (Life Technologies Corporation, Carlsbad, CA, USA). RNA integrity was assessed using the RNA Nano 6000 Assay Kit of the Agilent Bioanalyzer 2100 system (Agilent Technologies, Inc., Santa Clara, CA, USA). Oligo (dT) magnetic beads were used to enrich the mRNA, a fragmentation buffer was added to break mRNA into short fragments, and mRNA was used as the template to synthesize one-strand cDNA with random hexamers. After purifying the ds-cDNA, the ends were repaired, and the A tail and connecting adapter were added; then, AMPure XP beads were used for fragment size selection, and PCR was finally performed to obtain the cDNA library.

### 2.5. RNA-Seq Results Validation

The RT-qPCR primers for lipid differentiation marker genes, including *CEBPα*, *CEBPβ*, *SREBP1*, *PPARγ*, *Pref1*, and *LPL*, were synthesized by Beijing Qingke Biotechnology Co., LTD (Tsingke, Beijing, China) and are listed in Table 2. The relative expression of the above-mentioned genes was detected by RT-qPCR using the *UXT* gene as the reference gene.

### 2.6. Data Processing

The raw reads obtained from RNA-Seq were filtered to remove low-quality reads and those containing adapters, resulting in clean reads. The clean reads were then aligned to the goat reference genome using HISAT (v2.2.1) software, yielding mapped reads. Subsequently, gene expression levels for each sample were analyzed using HTSeq through a combined model. RPM (reads per million mapped reads) represents the number of reads per million mapped reads, while TPM (transcripts per million) refers to the number of mapped reads per kilobase of exon model transcript per million mapped reads. A threshold of 1 was set to determine whether a gene is expressed.

Raw sequencing reads were subjected to quality control using in-house Perl scripts provided by Novogene Co., Ltd. (Beijing, China). Reads containing adapter sequences, poly-N reads, and low-quality reads were removed to obtain clean reads. The resulting clean reads were aligned to the *Capra hircus* reference genome ARS1.2 (RefSeq accession: GCF_001704415.2; GenBank accession: GCA_001704415.2) using HISAT2 2.2.1, with gene annotation based on the NCBI RefSeq *Capra hircus* Annotation Release 102. Gene-level read counts were generated using HTSeq 2.0.5, and gene expression was quantified using RPM and/or TPM values as appropriate.

### 2.7. GO and KEGG

Count data were normalized and analyzed using DESeq, which models RNA-seq read counts based on a negative binomial distribution. Transcripts with |log_2_(Fold Change)| > 1.2 and adjusted *p*-value (*p*adj) < 0.05 were defined as differentially expressed mRNAs (DEmRNAs) and differentially expressed miRNAs (DEmiRNAs) between intramuscular fat (IMF) and subcutaneous fat (SCF) samples. Gene Ontology (GO) and Kyoto Encyclopedia of Genes and Genomes (KEGG) enrichment analyses were performed on DEmRNAs, as well as on the predicted target genes of DEmiRNAs.

For preliminary target gene prediction of DEmiRNAs, miRanda (v3.3a) software was employed based on sequence complementarity against the goat reference genome, with screening thresholds set as a match score ≥ 90 and minimum free energy ≤ −30 kcal/mol.

### 2.8. miRNA-mRNA Regulatory Network Construction

To construct a high-confidence miRNA-mRNA regulatory network, a two-step screening strategy was adopted. First, preliminary target gene prediction for all DEmiRNAs was performed using miRanda software with the same parameters described in Section 2.7. We retained miRNA-mRNA pairs that showed consistent differential expression trends between IMF and SCF for further filtering. Second, to improve the reliability of regulatory relationships, preliminary miRanda prediction results were intersected with target gene datasets from three public databases (TargetScan, miRDB, and miRTarBase). Only the overlapping miRNA–mRNA pairs were identified as high-confidence regulatory interactions. Finally, the screened regulatory pairs were imported into Cytoscape (v3.7.2) software for network visualization. Hub genes and key functional modules in the network were further analyzed, providing a system-level view of the core miRNA-mediated regulatory circuits underlying the functional differences between goat IMF and SCF.

Network topology was further analyzed based on degree centrality. The degree of a node was defined as the number of edges directly connected to that node. For an miRNA node, the degree value represented the number of candidate target mRNAs directly connected to the miRNA, whereas for an mRNA node, it represented the number of miRNAs connected to the mRNA. Nodes were ranked in descending order according to their degree values within each regulatory network, with a higher degree indicating a greater number of direct regulatory connections. Candidate hub miRNAs were identified based on their relative degree rankings in the corresponding networks. Other network topological measures, such as betweenness centrality and closeness centrality, were not evaluated in the present study. miRNAs with relatively high degree values were therefore described as high-degree hub nodes rather than being regarded as definitive core regulatory factors.

### 2.9. Function Verification of chi-miR-1

RT-qPCR primers for chi-miR-1, chi-miR-206, and chi-miR-127-3p were designed using the miRNA Design V1.01 software, and the primer sequences are listed in Table 3. The expression levels of these three DEmiRNAs were detected via RT-qPCR to assess the miRNA expression differences before and after the differentiation of both types of adipocytes.

Mimics and inhibitors of goat chi-miRNA-1 were synthesized by Shanghai Jima Biotechnology Co., Ltd. (Shanghai, China) and subsequently transfected into JNE and PE cells. JNE and PE cells were transfected using TurboFect Transfection Reagent (Thermo Fisher Scientific, Waltham, MA, USA; R0532). The final concentrations of both the chi-miR-1 mimic and chi-miR-1 inhibitor were 10 μM. Mimic negative control and inhibitor negative control groups were included at the same concentrations. After inducing differentiation for 3 days, the transfection efficiency was verified using RT-qPCR. Chi-miR-1 expression was normalized to the *UXT* gene, and its relative expression compared with the corresponding negative control group was calculated using the 2^−ΔΔCt^ method. Mimic transfection efficiency was evaluated based on the fold increase in chi-miR-1 expression, whereas inhibitor transfection efficiency was assessed based on the reduction in chi-miR-1 expression relative to the inhibitor negative control group.

Additionally, Oil Red O staining and BODIPY staining were introduced to observe the size and morphology of lipid droplets in both goat subcutaneous adipocytes and intramuscular adipocytes. Oil Red O staining was quantified by dissolving the retained dye in isopropanol and measuring absorbance at 490 nm. BODIPY staining was quantified by measuring the lipid droplet-positive area using ImageJ (v1.54t 16) software under identical image-acquisition and analysis settings across all experimental groups. Each experiment included five independently cultured cell replicates derived from the same donor animal, and five randomly selected fields were analyzed for each replicate.

### 2.10. Statistical Analysis

The obtained data were shown as “Mean ± SEM” and analyzed by 2^−ΔΔCt^. One-way analysis of variance in SPSS (v32.0.0) as used to detect the significance of the difference between the data. Data are presented as the mean ± SEM. Differences were considered statistically significant at *p* < 0.05 and highly significant at *p* < 0.01. Exact *p*-values are reported where available. For comparisons involving three or more groups, one-way ANOVA followed by Tukey’s multiple-comparison test was performed. Before ANOVA, data normality and homogeneity of variance were assessed using the Shapiro–Wilk test and Levene’s test, respectively. Differences were considered statistically significant at *p* < 0.05.

## 3. Results

### 3.1. Established a Cell Differentiation Model for Goat Intramuscular and Subcutaneous Pre-Adipocytes

**Figure 1:**
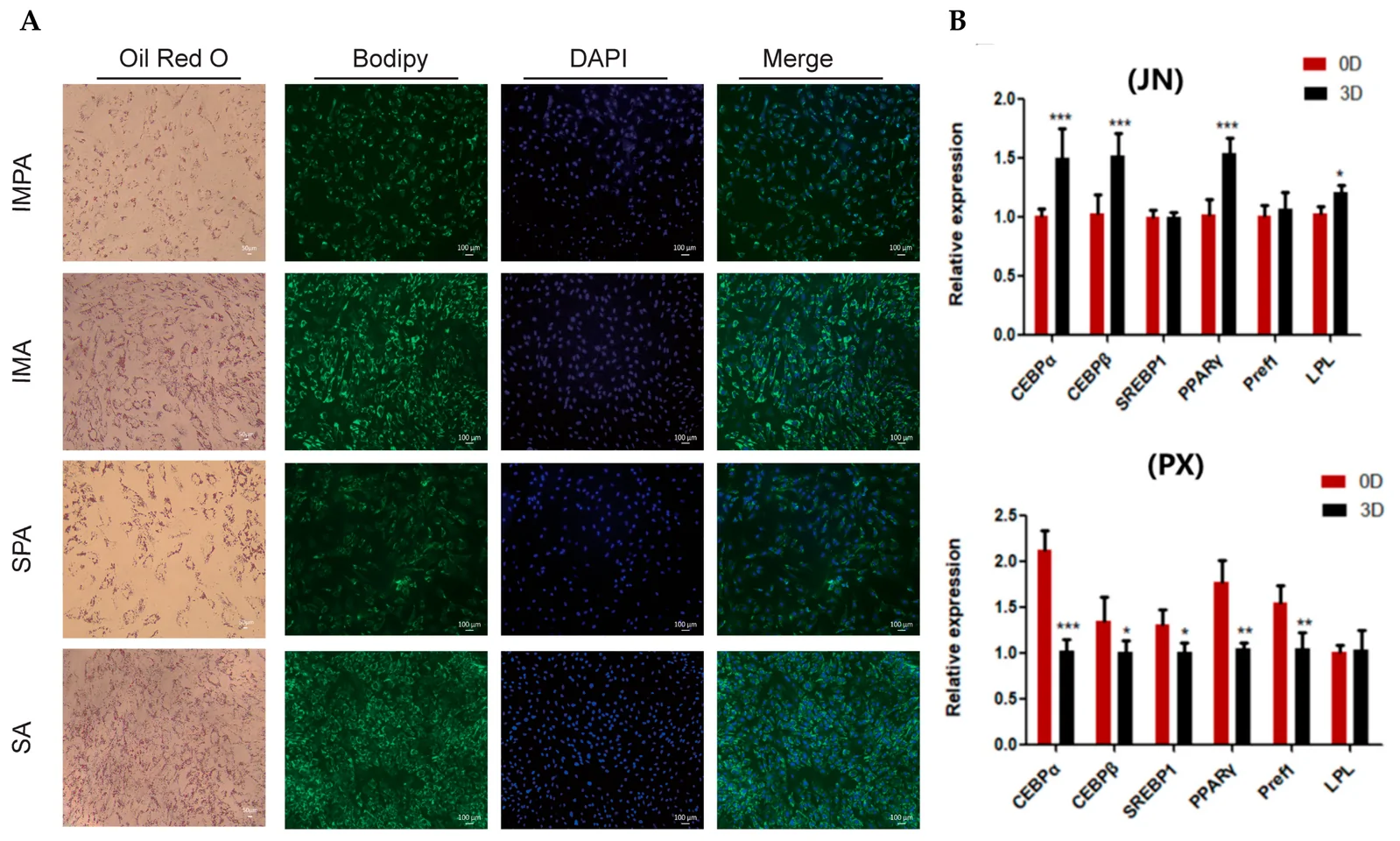
An in vitro cell differentiation model of preadipocytes from intramuscular fat and subcutaneous fat. (**A**) Representative Oil Red O staining (50×) and BODIPY staining (100×) images of intramuscular and subcutaneous cells before and after 72 h of adipogenic differentiation. SPA represents subcutaneous preadipocytes, SA represents subcutaneous adipocytes, IMPA represents intramuscular preadipocytes, and IMA represents intramuscular adipocytes. (**B**) Relative mRNA expression levels of adipocyte differentiation marker genes before and after 3-day differentiation induction in intramuscular preadipocytes (JN) and subcutaneous preadipocytes (PX). In intramuscular cells, *CEBPα*, *CEBPβ*, *PPARγ*, and *LPL* were significantly upregulated after differentiation, whereas no significant changes were observed in *SREBP1* or *Pref1*. In subcutaneous cells, *CEBPα*, *CEBPβ*, *SREBP1*, *PPARγ*, and *Pref1* were significantly downregulated after differentiation, whereas *LPL* showed no significant change. Data are presented as the mean ± SEM. “*” means *p* < 0.05, “**” means *p* < 0.01, and “***” means *p* < 0.001.

Following 72 h of adipogenic induction, both intramuscular and subcutaneous preadipocytes exhibited evident morphological changes and increased lipid droplet accumulation, as shown by Oil Red O and BODIPY staining (Figure 1A). RT-qPCR analysis further revealed distinct expression responses of adipogenic differentiation-related marker genes (Figure 1B). In intramuscular adipocytes, the expression levels of *CEBPα*, *CEBPβ*, and *PPARγ* were significantly increased after differentiation (*p* < 0.001), and *LPL* was also significantly upregulated (*p* < 0.05), whereas *SREBP1* and *Pref1* showed no significant changes. In subcutaneous adipocytes, *CEBPα* expression was significantly decreased (*p* < 0.001), while *CEBPβ* and *SREBP1* were decreased (*p* < 0.05), and *PPARγ* and *Pref1* were decreased (*p* < 0.01); no significant change was observed in *LPL* expression. Taken together, the morphological changes in lipid accumulation and the significant expression responses of adipogenic differentiation-related genes supported successful adipogenic differentiation of both intramuscular and subcutaneous preadipocytes.

### 3.2. Evaluation and Verification of Sequencing Data Quality

**Figure 2:**
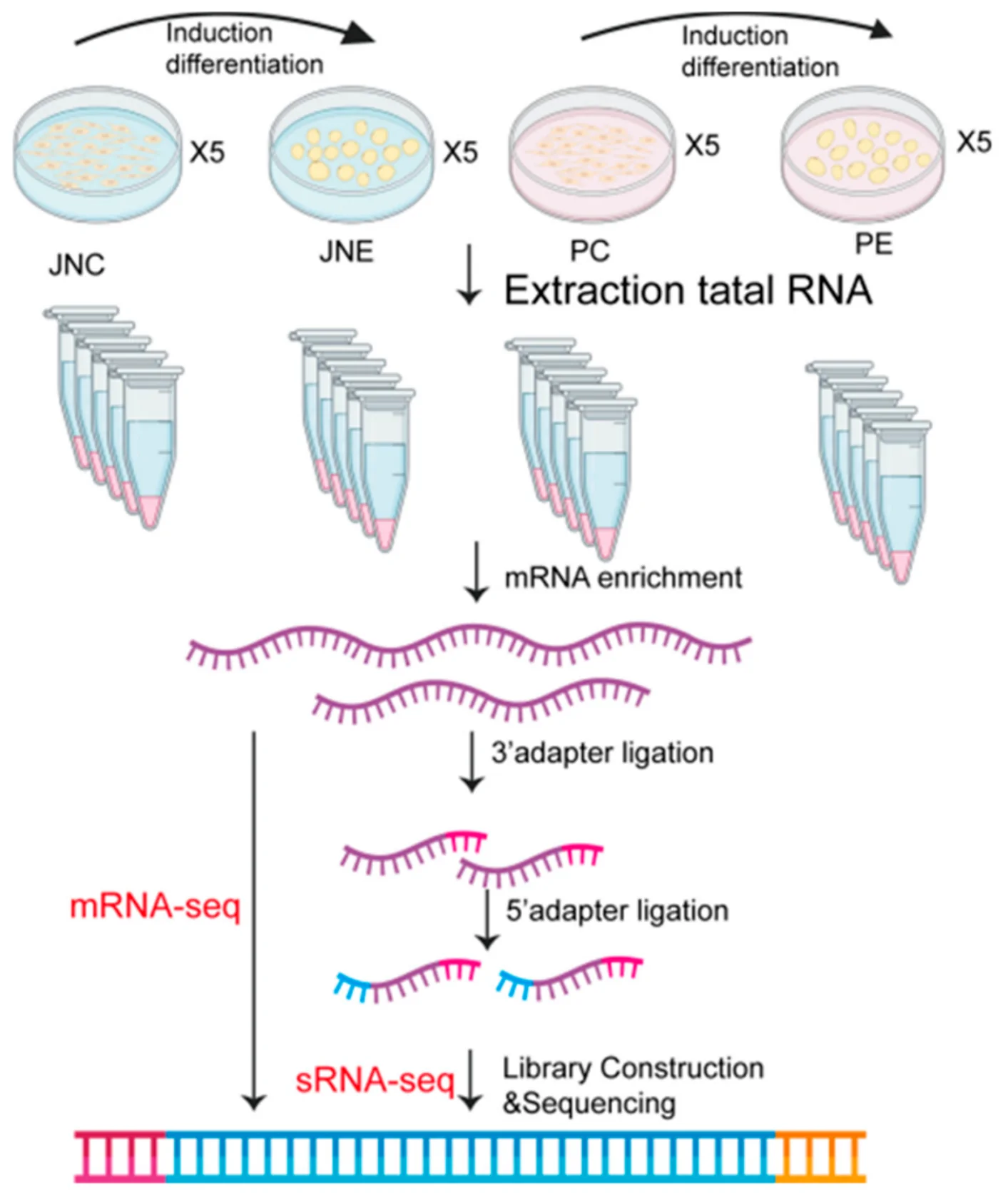
RNA sequencing flowchart.

**Figure 3:**
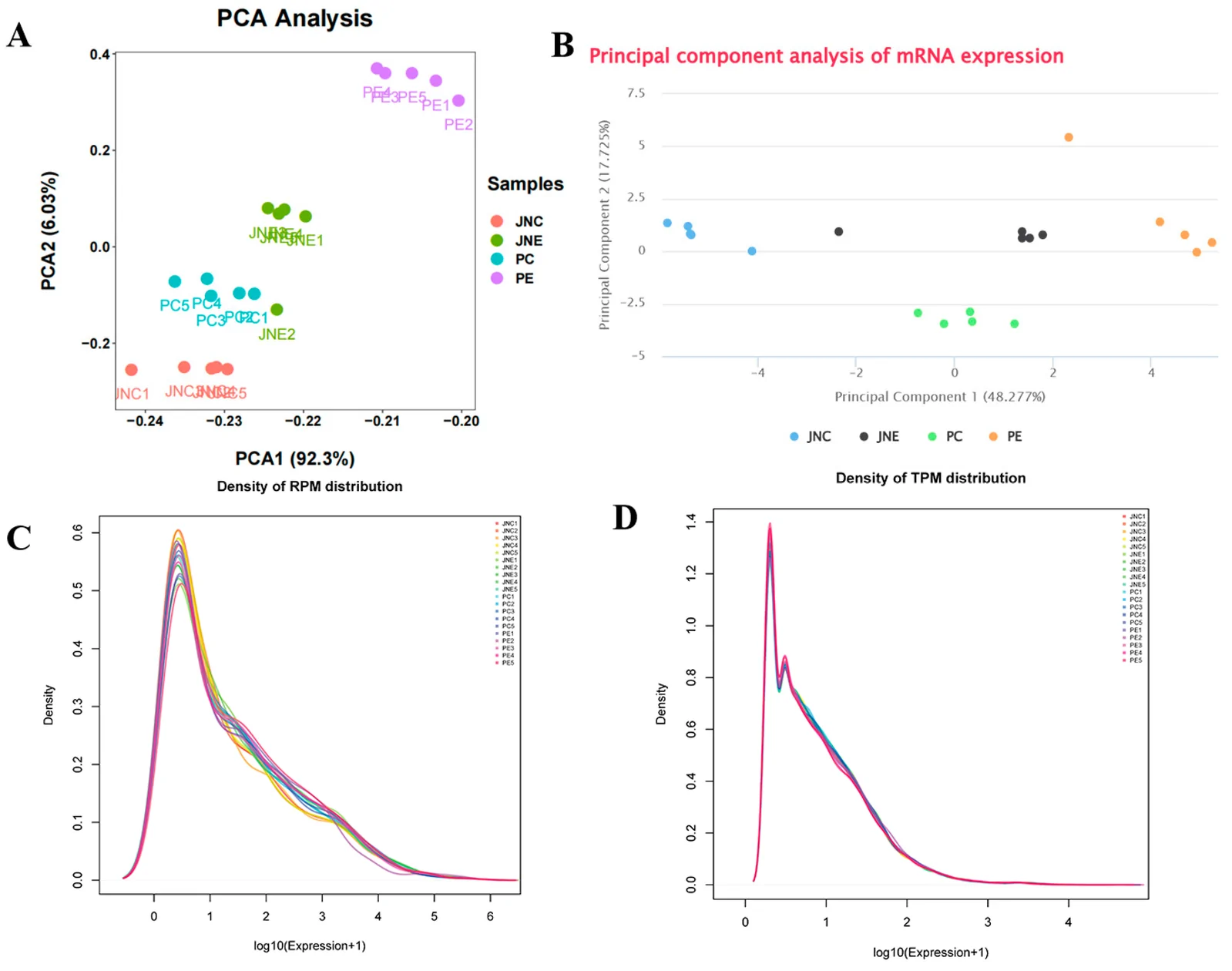
Characteristic analysis. (**A**) PCA analysis of miRNA expression; (**B**) PCA analysis of mRNA expression (Note: The y-axis values from bottom to top are −5, −2.5, 0, 2.5, 5, and 7.5.); (**C**) density of RPM distribution; (**D**) density of TPM distribution.

The RNA-seq sequencing procedure is shown in Figure 2. PCA and sample-correlation analyses showed that samples within the same experimental group exhibited similar expression profiles and tended to cluster together, whereas samples from different differentiation states or adipose depots showed distinct distribution patterns (Figure 3A,B). After quality filtering, 45.02–70.36 million clean reads were retained per library, with Q30 values ranging from 89.72% to 93.64%. The overall mapping rates to the goat reference genome ranged from 91.11% to 94.17%, of which 84.01–87.47% were uniquely mapped reads, while the multiple-mapping rates ranged from 6.70% to 7.96% (Table S1, Figure 3C,D). Collectively, the high sequencing quality, high mapping rates, and overall consistency among samples indicated that the sequencing datasets were of sufficient quality for subsequent differential expression and integrative analyses.

### 3.3. Differentially Expressed miRNAs and Functional Enrichment of Their Predicted Target Genes

**Figure 4:**
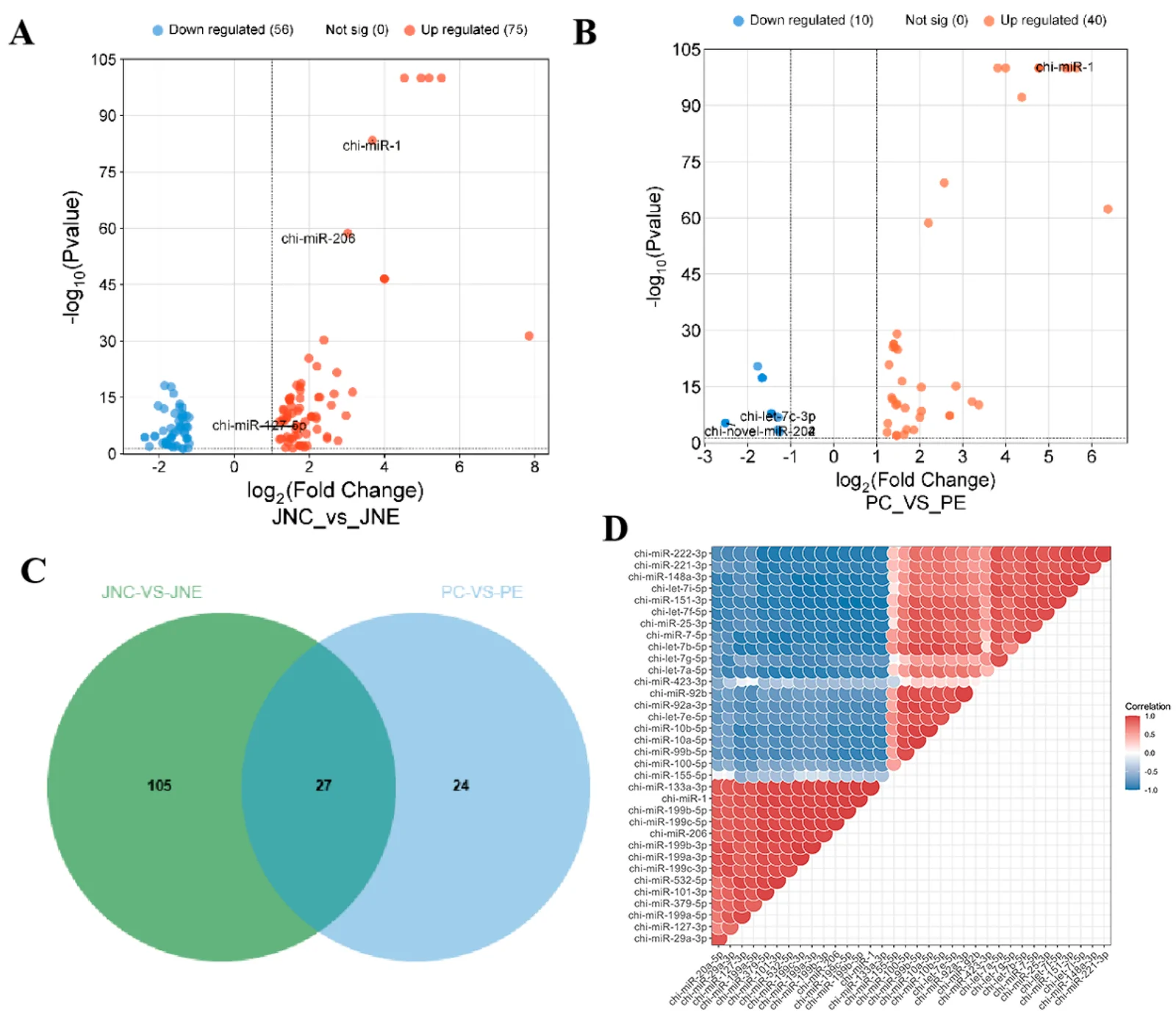
Expression profile of DEmiRNAs in intramuscular and subcutaneous adipocytes of goats. (**A**) Volcano plot of DEmiRNAs in intramuscular adipocytes; (**B**) volcano plot of DEmiRNAs in subcutaneous adipocytes; (**C**) Venn diagram of shared DEmiRNAs in intramuscular and subcutaneous adipocytes; (**D**) Representative DEmiRNAs. Note: JNE represents undifferentiated intramuscular preadipocytes, JNC represents differentiated intramuscular adipocytes, PE represents undifferentiated subcutaneous preadipocytes, and PC represents differentiated subcutaneous adipocytes.

In this study, DEmiRNAs before and after differentiation of goat intramuscular adipocytes and subcutaneous adipocytes were screened based on the following criteria: adjusted *p*-value < 0.05 and |Log2 (fold change)| > 1.2. A total of 131 DEmiRNAs were identified in goat intramuscular adipocytes, with 56 downregulated and 75 upregulated (Figure 4A). In goat subcutaneous adipocytes, 50 DEmiRNAs were identified, with 10 downregulated and 40 upregulated (Figure 4B). The intersection of DEmiRNAs between subcutaneous and intramuscular adipocytes revealed a common set of 27 shared DEmiRNAs (Figure 4C), and the representative DEmiRNAs are shown in Figure 4D.

**Figure 5:**
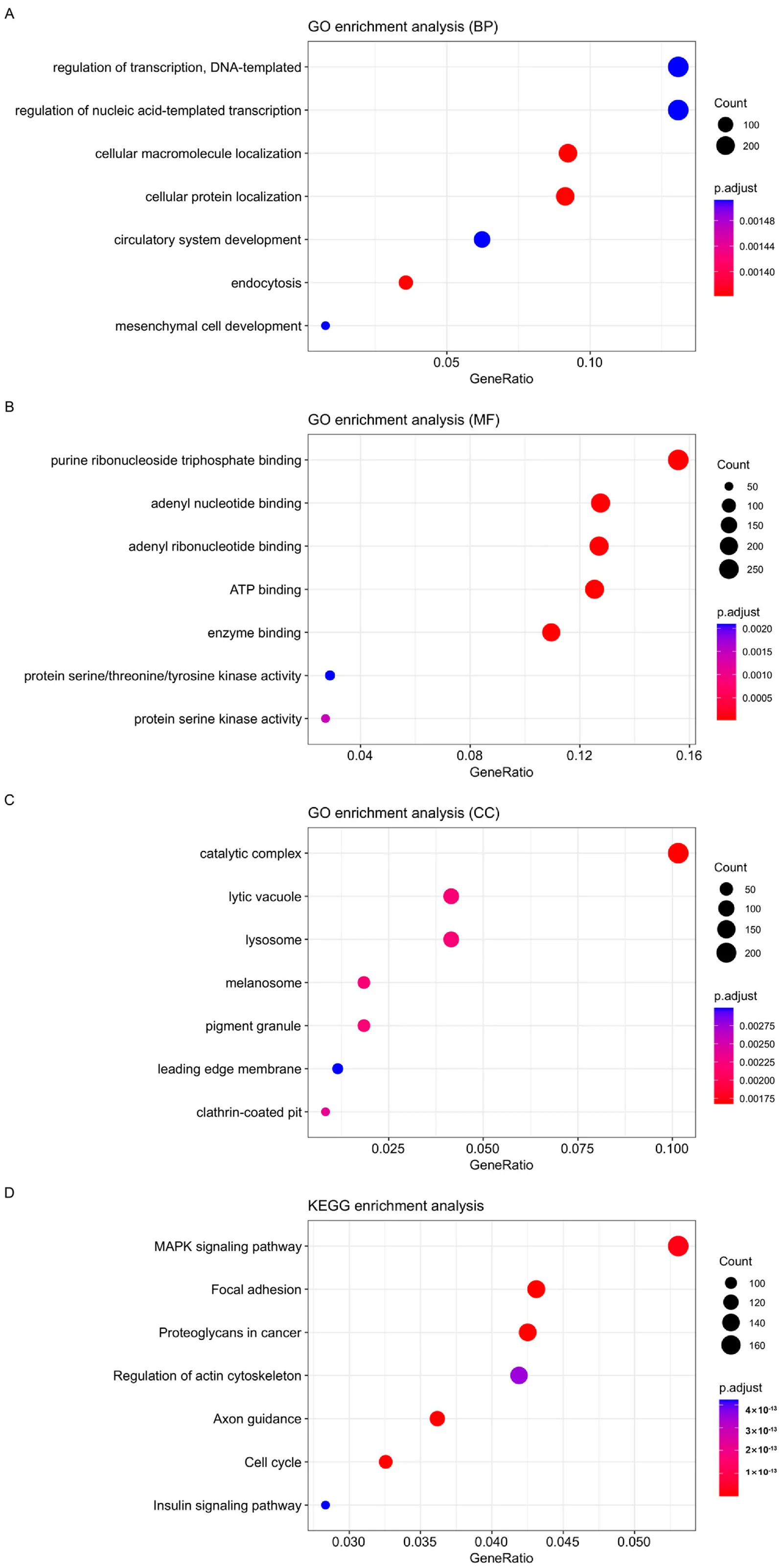
GO functional annotation and KEGG pathway enrichment analyses of the predicted target genes of differentially expressed miRNAs (DEmiRNAs). (**A**) The most enriched GO terms of biological process (BP); (**B**) the most enriched GO terms of molecular function (MF); (**C**) the most enriched GO terms of cellular component (CC); (**D**) Kyoto Encyclopedia of Genes and Genomes (KEGG) pathway enrichment analysis of the predicted target genes of DEmiRNAs. Dot size represents the number of genes enriched in each GO term or KEGG pathway, and dot color indicates the adjusted *p*-value.

To investigate the potential biological functions associated with the DEmiRNAs, GO functional annotation and KEGG pathway enrichment analyses were performed on their predicted target genes (Figure 5). GO enrichment analysis showed that the predicted target genes were mainly associated with biological processes, including transcription regulation, cellular protein localization, and cellular system development (Figure 5A). The most enriched molecular function terms included adenyl nucleotide binding, adenyl ribonucleotide binding, and purine ribonucleoside triphosphate binding (Figure 5B). For the cellular component category, the predicted target genes were mainly enriched in the catalytic complex, lytic vacuole, lysosome, melanosome, and pigment granule (Figure 5C). KEGG pathway enrichment analysis further revealed enrichment of the MAPK signaling pathway, focal adhesion, and cell cycle pathways (Figure 5D).

### 3.4. Differentially Expressed mRNAs and Functional Enrichment Analysis

**Figure 6:**
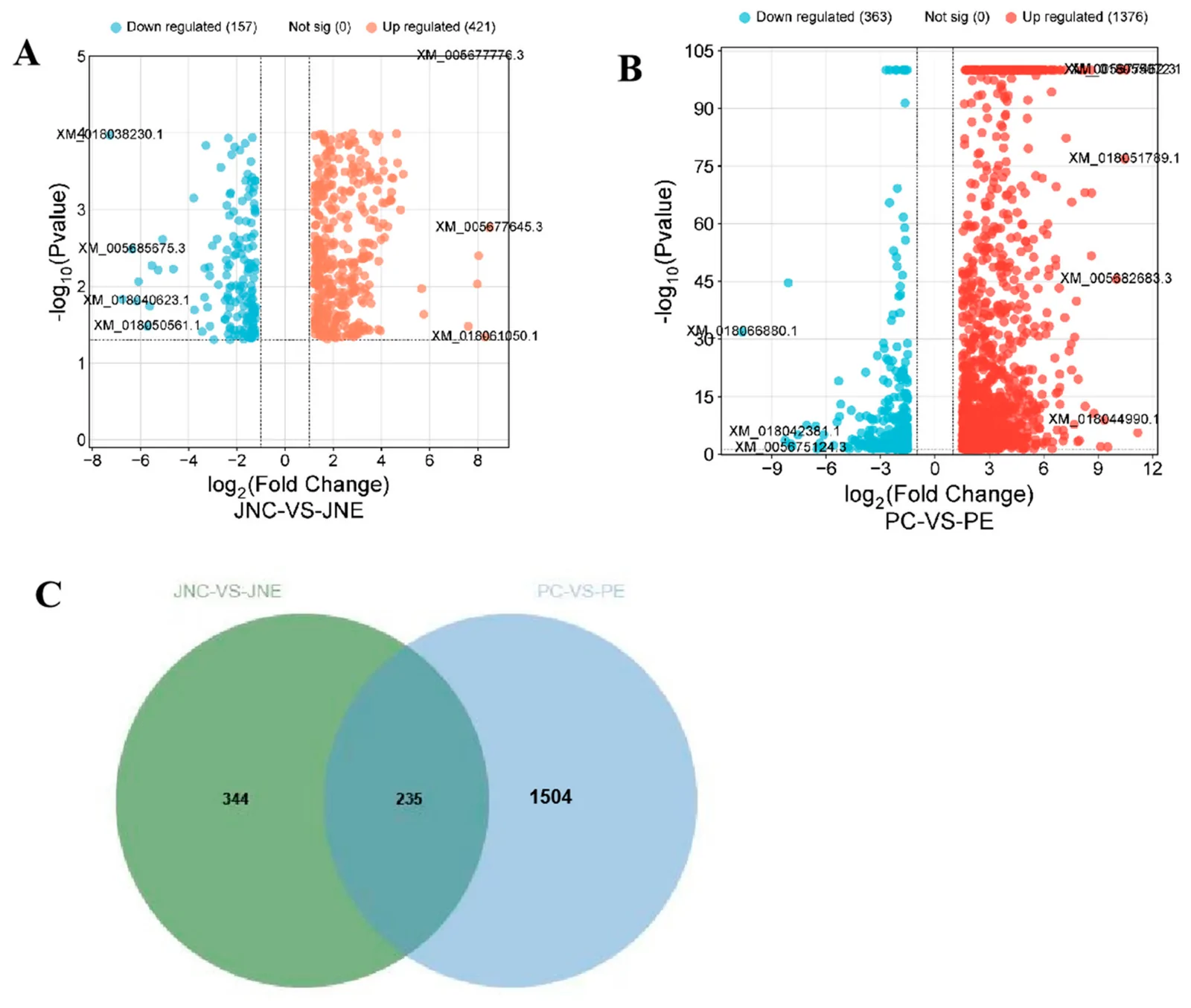
Expression profile of DEmRNAs in intramuscular and subcutaneous adipocytes of goats. (**A**) Volcano plot of DEmRNAs in intramuscular adipocytes; (**B**) volcano plot of DEmRNAs in subcutaneous adipocytes; (**C**) Venn diagram of shared DEmRNAs between subcutaneous and intramuscular adipocytes.

The DEmRNAs before and after differentiation of goat intramuscular adipocytes and subcutaneous adipocytes were screened based on the following criteria: adjusted *p*-value < 0.05 and |Log2 (fold change)| > 1.2. A total of 578 DEmRNAs were identified in goat intramuscular adipocytes, with 157 downregulated and 421 upregulated (Figure 6A). In goat subcutaneous adipocytes, 1739 DEmRNAs were identified, with 363 downregulated and 1376 upregulated (Figure 6B). The intersection of DEmRNAs between subcutaneous and intramuscular adipocytes revealed a common set of 235 shared DEmRNAs (Figure 6C).

**Figure 7:**
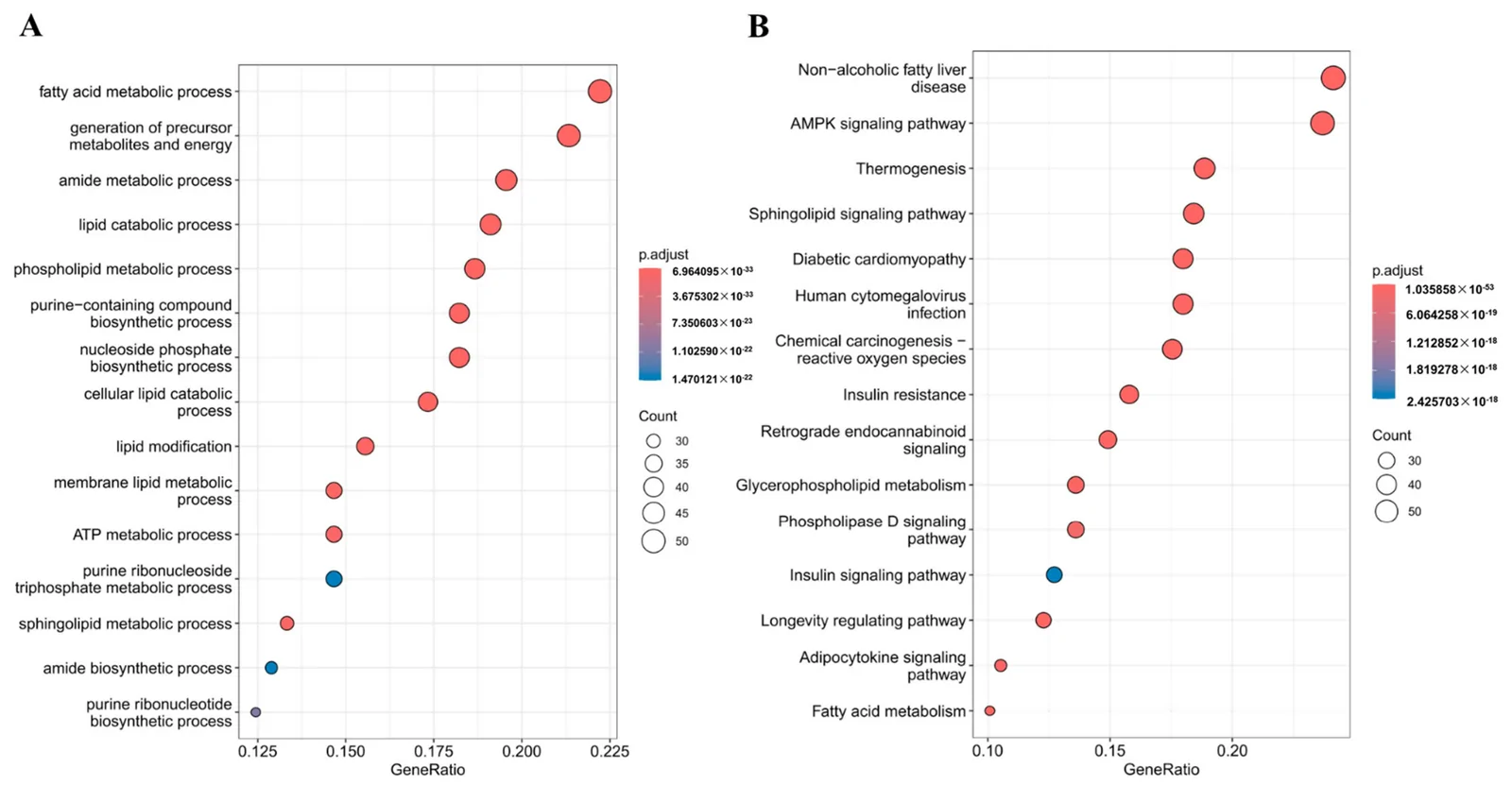
Functional enrichment analysis of differentially expressed mRNAs in goat intramuscular and subcutaneous adipocytes. (**A**) The most enriched GO terms of DEmRNAs; (**B**) enriched pathways of DEmRNAs. GeneRatio represents the proportion of DEmRNAs associated with each GO term or KEGG pathway relative to the total number of DEmRNAs included in the corresponding enrichment analysis. Dot size represents the number of DEmRNAs enriched in each GO term or KEGG pathway, and dot color indicates the adjusted *p*-value, with lower adjusted *p*-values representing greater enrichment significance.

To elucidate the regulatory roles of mRNAs in goat intramuscular and subcutaneous adipocytes, GO enrichment analysis and KEGG pathway analysis were performed on the identified DEmRNAs (Figure 7).

DEmRNAs were enriched mainly in the following GO terms: fatty acid process, lipid catabolic process, and lipid modification (Figure 7A). DEmRNAs were enriched in the AMPK signaling pathway, adipocytokine signaling pathway, and fatty acid metabolism (Figure 7B).

### 3.5. Construction of the miRNA–mRNA Regulatory Network for Adipocytes

**Figure 8:**
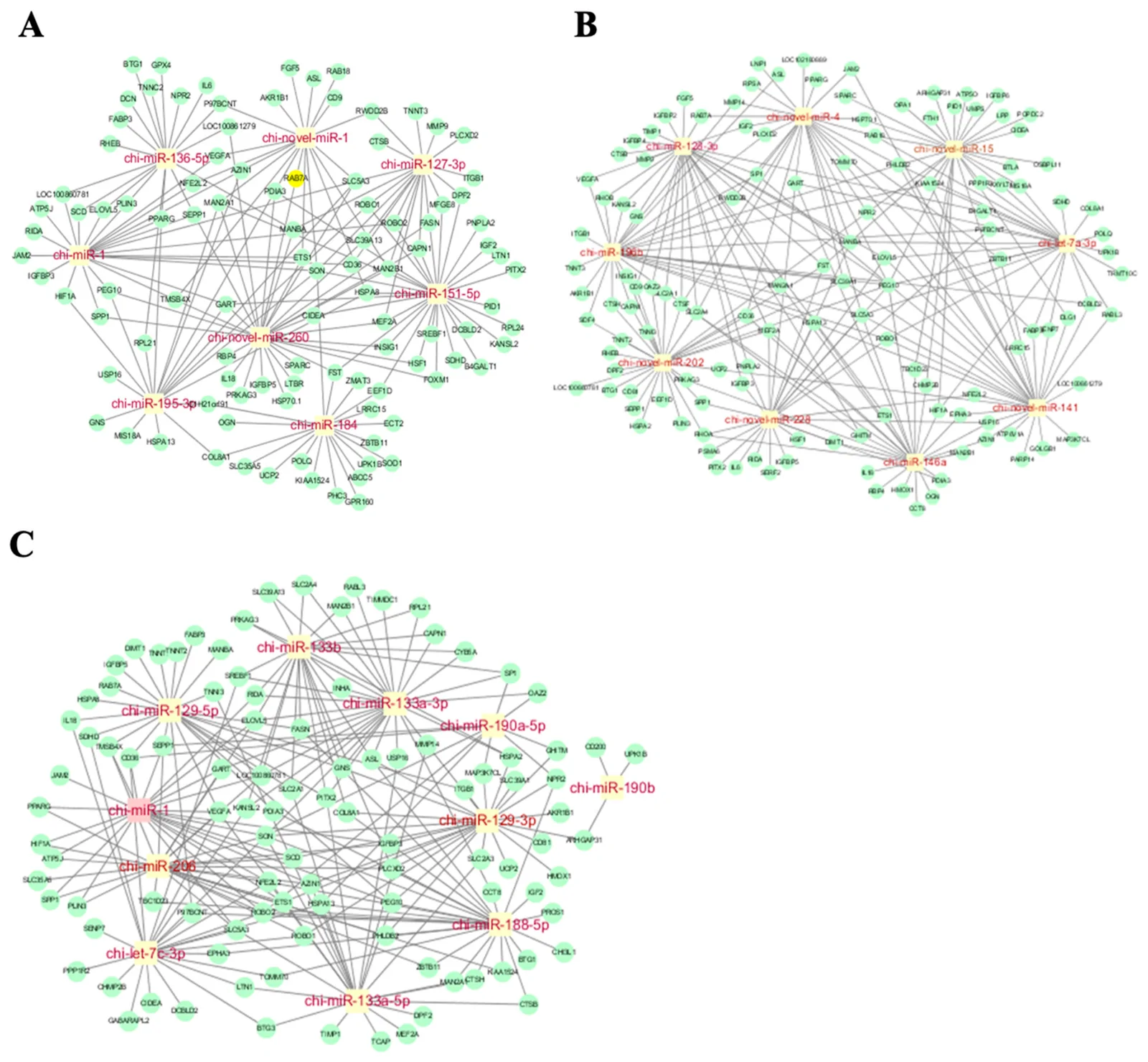
Predicted miRNA–mRNA regulatory networks for goat adipocytes. (**A**) miRNA–mRNA regulatory networks for goat intramuscular adipocytes; (**B**) miRNA–mRNA regulatory networks for goat subcutaneous adipocytes; (**C**) the shared miRNA–mRNA regulatory networks in goat intramuscular and subcutaneous adipocytes. Yellow nodes represent miRNAs, whereas green nodes represent their predicted candidate target mRNAs. Edges represent predicted miRNA–mRNA regulatory relationships retained after target prediction and network screening. Different node sizes were used to distinguish miRNA and mRNA nodes.

To identify the core regulators controlling adipogenesis in both IMF and SCF, we constructed miRNA-mRNA interaction networks for intramuscular adipocytes, subcutaneous adipocytes, and a shared network common to both (Figure 8A–C). The intramuscular adipocyte network comprised 110 nodes and 175 edges; the subcutaneous adipocyte network comprised 137 nodes and 244 edges; the shared network comprised 113 nodes and 232 edges, indicating a complex post-transcriptional regulatory landscape.

To pinpoint the most highly connected regulators within these networks, we performed topology analysis based on degree centrality. In the shared network, chi-miR-1 exhibited a degree value of 24, ranking first among the 11 miRNA nodes. Therefore, chi-miR-1 was identified as the highest-degree miRNA node in the shared regulatory network (Figure 8C). This bioinformatic discovery led us to hypothesize that chi-miR-1 might function as an important shared regulator of adipogenesis in both goat intramuscular and subcutaneous adipocytes.

Detailed information on all predicted miRNA–mRNA interaction pairs used to construct the intramuscular adipocyte, subcutaneous adipocyte, and shared regulatory networks is provided in Supplementary Tables S2–S4, respectively.

### 3.6. Functional Validation of chi-miR-1

**Figure 9:**
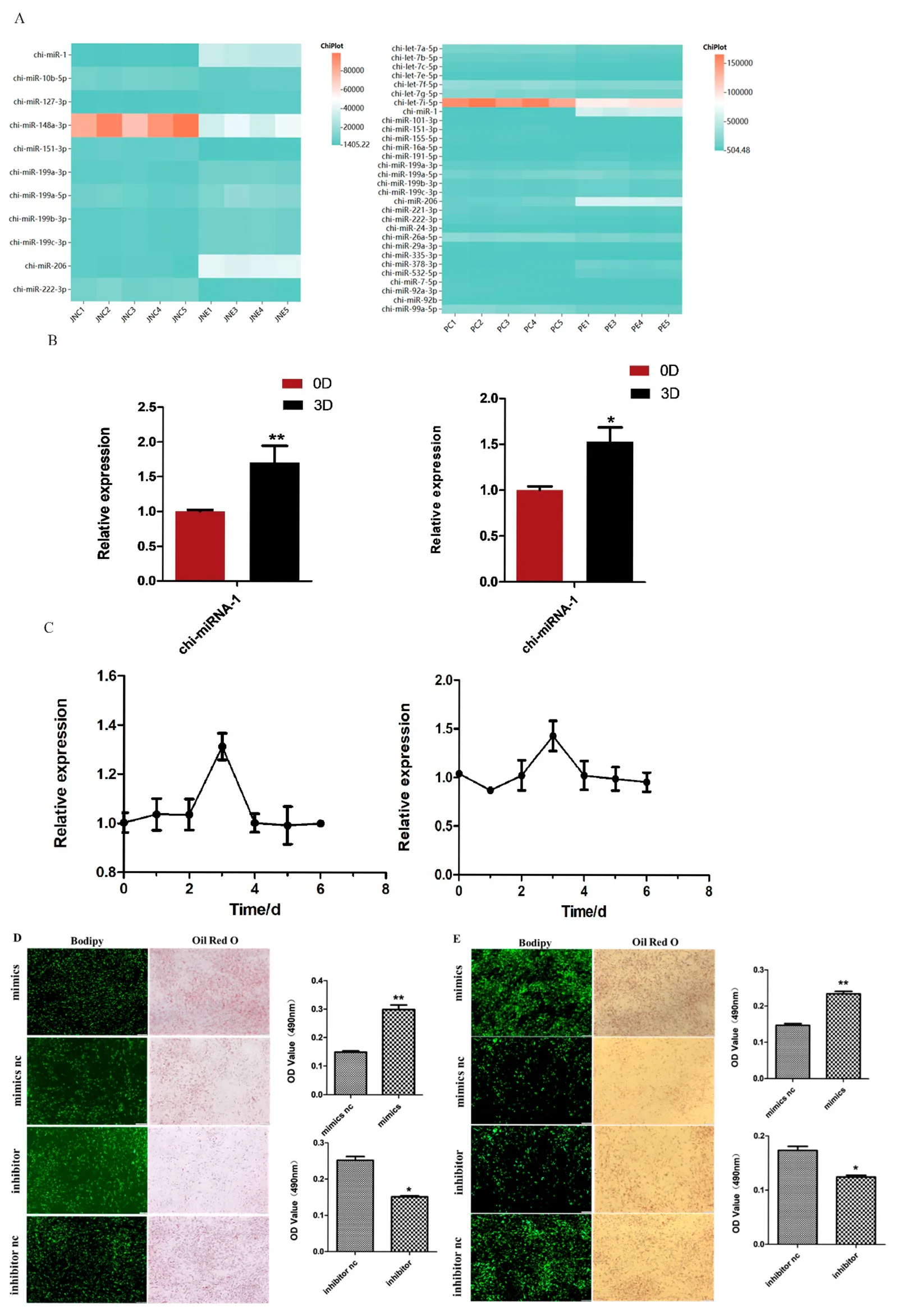
Functional validation of chi-miR-1 in goat intramuscular and subcutaneous adipocytes. (**A**) The top 10 DEmiRNAs in subcutaneous and intramuscular adipocytes. The left side shows intramuscular adipocytes, the right side shows subcutaneous adipocytes, and the same applies below. (**B**) The relative expression of chi-miRNA-1 before and after differentiation in intramuscular adipocytes (left panel) and subcutaneous adipocytes (right panel). (**C**) RT-qPCR verification of chi-miR-1 expression following transfection with the chi-miR-1 mimic, mimic negative control (mimic NC), chi-miR-1 inhibitor, or inhibitor negative control (inhibitor NC) in intramuscular adipocytes (left) and subcutaneous adipocytes (right). (**D**) Representative Oil Red O staining images (50×) and BODIPY staining images (100×), together with the corresponding quantitative analyses, in intramuscular adipocytes transfected with the chi-miR-1 mimic, mimic NC, chi-miR-1 inhibitor, or inhibitor NC. (**E**) Representative Oil Red O staining images (50×) and BODIPY staining images (100×), together with the corresponding quantitative analyses, in subcutaneous adipocytes subjected to the same treatments. Oil Red O staining was quantified by measuring the absorbance of the extracted dye at 490 nm, and BODIPY staining was quantified as the lipid droplet-positive area using ImageJ software. Each experiment included five independently cultured cell replicates derived from the same donor animal. For BODIPY quantification, five randomly selected fields were analyzed per replicate under identical image-acquisition and analysis settings. Data are presented as the mean ± SEM. Statistical significance was assessed using one-way ANOVA followed by Tukey’s multiple-comparison test. *p* < 0.05 (*) and *p* < 0.01 (**) versus the corresponding negative control.

Guided by the network analysis, we focused on validating the functional role of the hub miRNA, chi-miR-1. First, we confirmed that chi-miR-1 was significantly upregulated during the differentiation of both intramuscular and subcutaneous adipocytes (Figure 9A,B). To directly assess its function, we transfected preadipocytes with chi-miR-1 mimics and inhibitors and subsequently subjected them to adipogenic differentiation. Overexpression of chi-miR-1 significantly promoted lipid droplet accumulation, as evidenced by enhanced BODIPY and Oil Red O staining in both cell types (Figure 9C,D). Conversely, inhibition of chi-miR-1 impaired adipogenesis (Figure 9E). These results indicate that chi-miR-1 promotes the adipogenic differentiation of goat intramuscular and subcutaneous preadipocytes under the present in vitro conditions.

## 4. Discussion

With the development of high-throughput sequencing technology and the increasing number of bioinformatic analysis tools, more and more non-coding RNAs, including miRNA [16,17], lncRNA [18], and circRNA [19,20,21], were discovered and identified to participate in the process of animal fat deposition. In contrast, the study of shared potential key miRNAs and their target mRNAs that regulate adipogenesis in different fat tissues remains rare, especially in goats. The differentially expressed miRNAs and mRNAs before and after adipogenesis induction in goat IMF and SCF preadipocytes were analyzed, and the function and participating signaling pathways of these miRNAs were analyzed and enriched by GO and KEGG analysis. The miRNA-mRNA regulation networks in these two types of adipocyte cells were constructed in order to screen the key miRNAs and mRNAs in regulating the process of adipocyte cell differentiation. MiRNAs have been extensively investigated as regulators of lipogenesis [22], and it has been proved that, in many species, miRNAs are abundantly expressed in fat tissues and accompany the maturation process of adipocytes [23]. The MiRNA-mRNA regulation network has been shown to participate in many physiological functions in various domestic animals. For example, the miR-142-5p-FOXO3 axis influences skeletal muscle growth in chickens [24], the miR-218-5p-ACSL1 axis affects subcutaneous fat deposition in pigs [25], the miR-18-MEF2D axis influences the differentiation of skeletal muscle-derived satellite cells in beef cattle [26], and the miR-27a-PIK3R3 axis regulates the proliferation and apoptosis of hair follicle stem cells in sheep [27], indicating that miRNA-mRNA axis research has significant value in improving animal growth and meat quality.

High-throughput sequencing has discovered many key miRNAs in different tissues in goats, including miR-27 in goat mammary gland epithelial cells [28], miR-421 in goat intramuscular preadipocytes [29], and miR-133-3p in goat subcutaneous adipocytes [30]. The above-mentioned studies have suggested that the expression of miRNAs has tissue specificity. Thus, it is of great significance to reveal the key miRNAs in regulating fat deposition in different goat tissues.

The identified DEmiRNAs in the goat intramuscular adipocyte differentiation process, such as chi-miR-1197-3p, chi-miR-136-3p, chi-miR-184, and chi-miR-195-3p, have been reported in the goat brown adipose tissue whitening process [31]. This result strongly suggests that, although there are differences in physiological function and origin between intramuscular adipocytes and brown adipocytes, they may share certain key regulatory nodes in the differentiation/fate-determining molecular regulatory network, specifically these key miRNAs, and it is likely that these miRNAs function by targeting common key factors involved in adipocyte differentiation and cell fate determination. Also, these miRNAs may act at the intersection of key signaling pathways that regulate adipocyte differentiation and metabolism. For example, the AMPK signaling pathway not only affects intramuscular fat deposition [32] but also participates in the regulation of brown fat activity [33]. These miRNAs may produce similar or related effects in two different adipose biology processes by modulating the activity of these pathways. In goat subcutaneous adipocytes, several unreported miRNAs were identified, such as chi-miR-146a, chi-miR-196a, chi-miR-490, chi-miR-660, and some novel miRNAs, including chi-novel-miR-141, chi-novel-miR-15, chi-novel-miR-228, and chi-novel-miR-4. These findings may reveal species-specific or tissue-type-specific miRNAs in goat subcutaneous adipose tissue in regulating the adipogenesis process. The effect and the mechanism of these miRNAs on adipogenesis should be explored using an in vitro cell differentiation model.

Chi-miR-1 was previously reported to participate in hair follicle development in inner Mongolian cashmere goat [34] and was highly expressed in sheep skeletal muscle [35]. Our data demonstrated its potent pro-adipogenic role in goat intramuscular and subcutaneous adipocytes (Figure 9) by BODIPY staining, Oil red O staining, and qPCR. This functional antagonism suggests that chi-miR-1 may act as an important regulator and a potential molecular target for goat meat quality improvement since skeletal muscle and fat content determine the meat quality, though in vitro and in vivo validations of its spatiotemporal specificity are required.

We fully acknowledge that a major limitation of this study is that all intramuscular and subcutaneous preadipocytes were derived from a single donor animal. While we included five independently cultured cell replicates to assess the reproducibility of cellular responses, these replicates do not constitute independent biological replicates at the animal level. Consequently, our findings should be interpreted as donor-specific cellular responses rather than population-level norms, and future studies using primary cells derived from multiple genetically diverse animals, combined with in vivo models, are indispensable to validate the generality of the proposed miRNA–mRNA regulatory mechanisms.

## 5. Conclusions

In this study, we constructed miRNA-mRNA regulatory networks of adipocytes from different anatomical sites of a single goat donor. The results revealed several differentially expressed miRNAs shared in goat intramuscular and subcutaneous adipose tissues, including chi-miR-1 and chi-miR-206. Chi-miR-1 was found to promote adipogenic differentiation in both cell types under the present experimental conditions. However, these findings must be interpreted with caution given the single-animal origin of the cells, and the predicted interactions such as chi-miR-206–IGFBP3, chi-miR-133b–FASN, chi-miR-129-5p–IGFBP5, and chi-miR-1–PPARG, which may play roles in the process of adipogenesis, lipid metabolism, and lipid storage, should be regarded as candidate regulatory relationships that require further experimental validation.

## Figures and Tables

**Table 1:** Experimental design and grouping of goat adipocytes.

Groups	Adipose Tissue Site	Differentiation Status	Treatment and Sampling Time Point	Number of Replicates
JNE	Intramuscular adipose tissue	Undifferentiated preadipocytes	Before adipogenic induction, 0 h	5 cell culture replicates
JNC	Intramuscular adipose tissue	Differentiated adipocytes	After 72 h of adipogenic differentiation induced with 50 μmol/L oleic acid	5 cell culture replicates
PE	Subcutaneous adipose tissue	Undifferentiated preadipocytes	Before adipogenic induction, 0 h	5 cell culture replicates
PC	Subcutaneous adipose tissue	Differentiated adipocytes	After 72 h of adipogenic differentiation induced with 50 μmol/L oleic acid	5 cell culture replicates

**Table 2:** Primer sequence for RT-qPCR.

Gene Name	Sequence	Tm/°C	GenBank Accession Number	Product Length/bp
*CEBPα*	S: CCGTGGACAAGAACAGCAAC A: AGGCGGTCATTGTCACTGGT	58	XM_018062278	142
*CEBPβ*	S: CAAGAAGACGGTGGACAAGC A: AACAAGTTCCGCAGGGTG	66	XM_018058020.1	204
*SREBP1*	S: AAGTGGTGGGCCTCTCTGA A: GCAGGGGTTTCTCGGACT	58	NM_001285755	127
*PPARγ*	S: AAGCGTCAGGGTTCCACTATG A: GAACCTGATGGCGTTATGAGAC	60	NM_001285658	197
*Pref1*	S: CCGGCTTCATGGATAAGACCT A: GCCTCGCACTTGTTGAGGAA	65	KP686197.1	184
*LPL*	S: TCCTGGAGTGACGGAATCTGT A: GACAGCCAGTCCACCACGAT	60	NM_001285607	174
*UXT*	S: GCAAGTGGATTTGGGCTGTAAC A: ATGGAGTCCTTGGTGAGGTTGT	60	XM_005700842.2	180

**Table 3:** Primer information of DEmiRNAs.

Gene Name	RT-qPCR Primer Sequence	Tm/°C	RT-PCR Primer Sequence	Tm/°C
*chi-miR-1*	S: GCGCGTGGAATGTAAAGAAGT A: AGTGCAGGGTCCGAGGTATT	59	5′--GTCGTATCCAGTGCAGGGTCCGAGGTATTCGCACTGGATACGACATACAT--3′	60
*chi-miR-206*	S: CGCGTGGAATGTAAGGAAGTG A: AGTGCAGGGTCCGAGGTATT	60	5′--GTCGTATCCAGTGCAGGGTCCGAGGTATTCGCACTGGATACGACACCACA--3′	60
*chi-miR-127-3p*	S: CGCGTCGGATCCGTCTGA A: AGTGCAGGGTCCGAGGTATT	61	5′--GTCGTATCCAGTGCAGGGTCCGAGGTATTCGCACTGGATACGACCCAAGC--3′	60

## Data Availability

The datasets generated and analyzed during the current study include raw mRNA and miRNA sequencing data, processed gene and miRNA expression matrices, differential expression results, predicted miRNA–mRNA regulatory relationships, and data from the functional validation experiments. These datasets have not yet been deposited in a public repository and are available from the corresponding author upon reasonable request.

## Supplementary Materials

The following supporting information can be downloaded at https://www.mdpi.com/article/10.3390/ani16162603/s1. Table S1: RNA-seq quality filtering; Table S2: miRNA–mRNA interaction pairs in the intramuscular adipocyte regulatory network. title; Table S3: miRNA–mRNA interaction pairs in the subcutaneous adipocyte regulatory network; Table S4: miRNA–mRNA interaction pairs in the shared regulatory network.

## Abbreviations

**Item**	**Definition**
IMF	Intramuscular fat
SCF	Subcutaneous fat
DEmiRNAs	Differentially expressed miRNAs
MAPKs	Mitogen-activated protein kinases
Wnt	Wingless/integrated
3ʹUTR	3ʹ-untranslated region
circRNAs	Circular RNAs
lncRNAs	Long non-coding RNAs
JH	Jinhua
LD	Landrace
PCA	Principal component analysis
GO	Gene Ontology
KEGG	Kyoto Encyclopedia of Genes and Genomes
BP	Biological process
MF	Molecular function
CC	Cellular component
AMPK	AMP-activated protein kinase
CEBPα	CCAAT enhancer binding protein alpha
CEBPβ	CCAAT enhancer binding protein beta
SREBP1	Sterol regulatory element binding protein-1
PPARγ	Peroxisome proliferator-activated receptor, gamma
Pref1	Preadipocyte factor 1
LPL	Lipoprotein lipase
RPM	Reads per million mapped reads
TPM	Transcripts per million
