# Supplementary material for: Identification of miRNA-mRNA Regulatory Networks Reveals Potential Key miRNAs in Goat Intramuscular and Subcutaneous Adipose Formation
Source: Animals (Basel). 2026 Aug 20;16(16):2603. doi: 10.3390/ani16162603 (PMC13508972; doi:10.3390/ani16162603)
Supplement: Supplementary file 1 [file animals-16-02603-s001.zip › Supplementary Table S1.pdf]

Supplementary Table S1.

**Sample**	**Raw reads**	**Clean reads**	**Clean bases(G)**	**Q20(%)**	**Q30(%)**	**GC(%)**	**Total mapped**	**Multiple mapped**	**Uniquely mapped**
JNC1	70994874	67935994	10.19	97.25	93.06	52.94	62953150 (92.67%)	5237923 (7.71%)	57715227 (84.96%)
JNC2	74476968	70360748	10.55	95.49	89.72	51.4	64102625 (91.11%)	4995413 (7.1%)	59107212 (84.01%)
JNC3	54696246	52516798	7.88	97.28	93.07	51.9	49141836 (93.57%)	3694920 (7.04%)	45446916 (86.54%)
JNC4	46981052	45019032	6.75	97.13	92.77	51.49	42149401 (93.63%)	3192278 (7.09%)	38957123 (86.53%)
JNC5	48522720	46431818	6.96	97.02	92.55	52.17	43326945 (93.31%)	3411261 (7.35%)	39915684 (85.97%)
JNE1	53375976	51226382	7.68	97.12	92.72	51.75	47894120 (93.5%)	3725963 (7.27%)	44168157 (86.22%)
JNE2	55647748	53381458	8.01	97.12	92.75	52.18	49786851 (93.27%)	3939514 (7.38%)	45847337 (85.89%)
JNE3	56587750	54317774	8.15	97.25	92.98	51.91	50808906 (93.54%)	4087712 (7.53%)	46721194 (86.01%)
JNE4	57404076	54950904	8.24	97.18	92.85	51.65	51358043 (93.46%)	4086507 (7.44%)	47271536 (86.03%)
JNE5	49316340	47363238	7.1	96.94	92.17	51.42	44437474	3574353	40863121

							(93.82%)	(7.55%)	(86.28%)
PC1	55474220	53373882	8.01	97.4	93.31	50.98	50263159 (94.17%)	3577129 (6.7%)	46686030 (87.47%)
PC2	52821766	50684288	7.6	97.23	92.96	51.24	47587314 (93.89%)	3476959 (6.86%)	44110355 (87.03%)
PC3	57973970	55633750	8.35	97.17	92.8	51.39	52180500 (93.79%)	3970332 (7.14%)	48210168 (86.66%)
PC4	55536832	53400554	8.01	97.29	93.04	52.38	49915741 (93.47%)	3875823 (7.26%)	46039918 (86.22%)
PC5	54717942	52603320	7.89	97.35	93.17	51.96	49283338 (93.69%)	3841692 (7.3%)	45441646 (86.39%)
PE1	59566694	57311574	8.6	97.33	93.13	51.93	53701324 (93.7%)	4453868 (7.77%)	49247456 (85.93%)
PE2	57061488	54454502	8.17	97.31	93.1	52.3	51276553 (94.16%)	3974520 (7.3%)	47302033 (86.87%)
PE3	50873478	48907340	7.34	97.54	93.64	52.4	45876242 (93.8%)	3665410 (7.49%)	42210832 (86.31%)
PE4	63681976	61450164	9.22	97.12	92.78	52.59	57344207 (93.32%)	4769600 (7.76%)	52574607 (85.56%)
PE5	60708268	58310358	8.75	97.33	93.2	53.13	54273672 (93.08%)	4642581 (7.96%)	49631091 (85.12%)
