# Supplementary material for: Identification of miRNA-mRNA Regulatory Networks Reveals Potential Key miRNAs in Goat Intramuscular and Subcutaneous Adipose Formation
Source: Animals (Basel). 2026 Aug 20;16(16):2603. doi: 10.3390/ani16162603 (PMC13508972; doi:10.3390/ani16162603)
Supplement: Supplementary file 1 [file animals-16-02603-s001.zip › Supplementary Table S2. miRNA–mRNA interaction pairs in the intramuscular adipocyte regulatory network..pdf]

Supplementary Table S2. miRNA–mRNA interaction pairs in the intramuscular adipocyte regulatory network.

miRNA	Target mRNA
chi-miR-127-3p	CD36
chi-miR-127-3p	DPF2
chi-miR-127-3p	FASN
chi-miR-127-3p	CAPN1
chi-miR-127-3p	MANBA
chi-miR-127-3p	SLC39A13
chi-miR-127-3p	ITGB1
chi-miR-127-3p	TNNT3
chi-miR-127-3p	MEF2A
chi-miR-127-3p	CTSB
chi-miR-127-3p	MMP9
chi-miR-127-3p	MFGE8
chi-miR-127-3p	MAN2B1
chi-miR-127-3p	SLC5A3
chi-miR-127-3p	SON
chi-miR-127-3p	RWDD2B
chi-miR-127-3p	ROBO2
chi-miR-127-3p	ROBO1
chi-miR-127-3p	PLCXD2
chi-miR-136-5p	DCN
chi-miR-136-5p	SEPP1
chi-miR-136-5p	PLIN3
chi-miR-136-5p	SCD
chi-miR-136-5p	LOC100861279
chi-miR-136-5p	ELOVL5
chi-miR-136-5p	RPL21
chi-miR-136-5p	LOC100860781
chi-miR-136-5p	IL6
chi-miR-136-5p	RHEB
chi-miR-136-5p	PPARG
chi-miR-136-5p	TNNC2
chi-miR-136-5p	NPR2
chi-miR-136-5p	BTG1
chi-miR-136-5p	MANBA
chi-miR-136-5p	P97BCNT
chi-miR-136-5p	FABP3
chi-miR-136-5p	GPX4
chi-miR-184	SOD1
chi-miR-184	OGN

chi-miR-184	FST
chi-miR-184	UCP2
chi-miR-184	EEF1D
chi-miR-184	SON
chi-miR-184	C1H21orf91
chi-miR-184	COL8A1
chi-miR-184	ZBTB11
chi-miR-184	KIAA1524
chi-miR-184	SLC35A5
chi-miR-184	UPK1B
chi-miR-184	POLQ
chi-miR-184	LRRC15
chi-miR-184	ABCC5
chi-miR-184	ZMAT3
chi-miR-184	ECT2
chi-miR-184	GPR160
chi-miR-184	PHC3
chi-miR-195-3p	RPL21
chi-miR-195-3p	GNS
chi-miR-195-3p	HIF1A
chi-miR-195-3p	PDIA3
chi-miR-195-3p	MAN2A1
chi-miR-195-3p	PEG10
chi-miR-195-3p	AZIN1
chi-miR-195-3p	HSPA8
chi-miR-195-3p	SLC5A3
chi-miR-195-3p	GART
chi-miR-195-3p	SON
chi-miR-195-3p	MIS18A
chi-miR-195-3p	USP16
chi-miR-195-3p	C1H21orf91
chi-miR-195-3p	HSPA13
chi-miR-195-3p	ROBO1
chi-miR-195-3p	COL8A1
chi-miR-195-3p	PPARG
chi-novel-miR-1	CD9
chi-novel-miR-1	P97BCNT
chi-novel-miR-1	ASL
chi-novel-miR-1	PDIA3
chi-novel-miR-1	ETS1
chi-novel-miR-1	RAB7A
chi-novel-miR-1	TMSB4X
chi-novel-miR-1	VEGFA

chi-novel-miR-1	RAB18
chi-novel-miR-1	MAN2A1
chi-novel-miR-1	AZIN1
chi-novel-miR-1	FGF5
chi-novel-miR-1	HSPA8
chi-novel-miR-1	AKR1B1
chi-novel-miR-1	NFE2L2
chi-novel-miR-1	MAN2B1
chi-novel-miR-1	SLC5A3
chi-novel-miR-1	SON
chi-novel-miR-1	RWDD2B
chi-miR-151-5p	DPF2
chi-miR-151-5p	FASN
chi-miR-151-5p	CAPN1
chi-miR-151-5p	SDHD
chi-miR-151-5p	PDIA3
chi-miR-151-5p	PNPLA2
chi-miR-151-5p	SREBF1
chi-miR-151-5p	FST
chi-miR-151-5p	B4GALT1
chi-miR-151-5p	INSIG1
chi-miR-151-5p	KANSL2
chi-miR-151-5p	IGF2
chi-miR-151-5p	MAN2A1
chi-miR-151-5p	CIDEA
chi-miR-151-5p	EEF1D
chi-miR-151-5p	MEF2A
chi-miR-151-5p	HSPA8
chi-miR-151-5p	PITX2
chi-miR-151-5p	MFGE8
chi-miR-151-5p	FOXM1
chi-miR-151-5p	MAN2B1
chi-miR-151-5p	HSF1
chi-miR-151-5p	PID1
chi-miR-151-5p	GART
chi-miR-151-5p	SON
chi-miR-151-5p	LTN1
chi-miR-151-5p	ROBO2
chi-miR-151-5p	ROBO1
chi-miR-151-5p	RPL24
chi-miR-151-5p	DCBLD2
chi-novel-miR-260	IL18
chi-novel-miR-260	IGFBP5

chi-novel-miR-260	SEPP1
chi-novel-miR-260	LOC100861279
chi-novel-miR-260	FASN
chi-novel-miR-260	PRKAG3
chi-novel-miR-260	SPARC
chi-novel-miR-260	CAPN1
chi-novel-miR-260	SPP1
chi-novel-miR-260	MANBA
chi-novel-miR-260	HSP70.1
chi-novel-miR-260	PDIA3
chi-novel-miR-260	FST
chi-novel-miR-260	ETS1
chi-novel-miR-260	RAB7A
chi-novel-miR-260	SLC39A13
chi-novel-miR-260	TMSB4X
chi-novel-miR-260	MAN2A1
chi-novel-miR-260	PEG10
chi-novel-miR-260	CIDEA
chi-novel-miR-260	RBP4
chi-novel-miR-260	MEF2A
chi-novel-miR-260	LTBR
chi-novel-miR-260	FOXM1
chi-novel-miR-260	MAN2B1
chi-novel-miR-260	HSF1
chi-novel-miR-260	SLC5A3
chi-novel-miR-260	GART
chi-novel-miR-260	INSIG1
chi-miR-1	CD36
chi-miR-1	SEPP1
chi-miR-1	PLIN3
chi-miR-1	SCD
chi-miR-1	ELOVL5
chi-miR-1	LOC100860781
chi-miR-1	PPARG
chi-miR-1	SPP1
chi-miR-1	RIDA
chi-miR-1	HIF1A
chi-miR-1	ETS1
chi-miR-1	TMSB4X
chi-miR-1	VEGFA
chi-miR-1	PEG10
chi-miR-1	AZIN1
chi-miR-1	IGFBP3

chi-miR-1	NFE2L2
chi-miR-1	SLC5A3
chi-miR-1	GART
chi-miR-1	SON
chi-miR-1	ATP5J
chi-miR-1	JAM2
chi-miR-1	ROBO2
