# Supplementary material for: Identification of miRNA-mRNA Regulatory Networks Reveals Potential Key miRNAs in Goat Intramuscular and Subcutaneous Adipose Formation
Source: Animals (Basel). 2026 Aug 20;16(16):2603. doi: 10.3390/ani16162603 (PMC13508972; doi:10.3390/ani16162603)
Supplement: Supplementary file 1 [file animals-16-02603-s001.zip › Supplementary Table S3. miRNA–mRNA interaction pairs in the subcutaneous adipocyte regulatory network..pdf]

Supplementary Table S3. miRNA–mRNA interaction pairs in the subcutaneous adipocyte regulatory network.

miRNA	Target mRNA
chi-novel-miR-4	PEG10
chi-novel-miR-4	PHLDB2
chi-novel-miR-4	PLCXD2
chi-novel-miR-4	ZBTB11
chi-novel-miR-4	LOC102180889
chi-novel-miR-4	LNP1
chi-novel-miR-4	TOMM70
chi-novel-miR-4	JAM2
chi-novel-miR-4	RWDD2B
chi-novel-miR-4	GART
chi-novel-miR-4	SLC5A3
chi-novel-miR-4	CTSF
chi-novel-miR-4	SLC2A4
chi-novel-miR-4	RAB18
chi-novel-miR-4	IGF2
chi-novel-miR-4	RAB7A
chi-novel-miR-4	MMP14
chi-novel-miR-4	SP1
chi-novel-miR-4	RPSA
chi-novel-miR-4	HSP70.1
chi-novel-miR-4	FABP3
chi-novel-miR-4	MANBA
chi-novel-miR-4	NPR2
chi-novel-miR-4	SLC39A1
chi-novel-miR-4	PPARG
chi-novel-miR-4	CD36
chi-novel-miR-202	PEG10
chi-novel-miR-202	CTSF
chi-novel-miR-202	HSPA2
chi-novel-miR-202	TNNI3
chi-novel-miR-202	MEF2A
chi-novel-miR-202	SLC2A4
chi-novel-miR-202	EEF1D
chi-novel-miR-202	SLC2A1
chi-novel-miR-202	TNNT2
chi-novel-miR-202	CTSH
chi-novel-miR-202	UCP2
chi-novel-miR-202	OAZ2
chi-novel-miR-202	RHOA

chi-novel-miR-202	INSIG1
chi-novel-miR-202	FST
chi-novel-miR-202	BTG1
chi-novel-miR-202	CD81
chi-novel-miR-202	NPR2
chi-novel-miR-202	CD9
chi-novel-miR-202	SPP1
chi-novel-miR-202	SLC39A1
chi-novel-miR-202	CAPN1
chi-novel-miR-202	RHEB
chi-novel-miR-202	PRKAG3
chi-novel-miR-202	SDF4
chi-novel-miR-202	LOC100860781
chi-novel-miR-202	DPF2
chi-novel-miR-202	PLIN3
chi-novel-miR-202	SEPP1
chi-novel-miR-202	CD36
chi-novel-miR-228	PEG10
chi-novel-miR-228	ROBO1
chi-novel-miR-228	HSPA13
chi-novel-miR-228	USP16
chi-novel-miR-228	GART
chi-novel-miR-228	SLC5A3
chi-novel-miR-228	HSF1
chi-novel-miR-228	NFE2L2
chi-novel-miR-228	PITX2
chi-novel-miR-228	MEF2A
chi-novel-miR-228	IGFBP3
chi-novel-miR-228	SERF2
chi-novel-miR-228	RHOA
chi-novel-miR-228	FST
chi-novel-miR-228	PNPLA2
chi-novel-miR-228	PDIA3
chi-novel-miR-228	RIDA
chi-novel-miR-228	PSMA6
chi-novel-miR-228	P97BCNT
chi-novel-miR-228	MANBA
chi-novel-miR-228	SPP1
chi-novel-miR-228	IL6
chi-novel-miR-228	ELOVL5
chi-novel-miR-228	DIMT1
chi-novel-miR-228	IGFBP5
chi-novel-miR-15	PEG10

chi-novel-miR-15	LPP
chi-novel-miR-15	OPA1
chi-novel-miR-15	LRRC15
chi-novel-miR-15	XXYLT1
chi-novel-miR-15	PPP1R2
chi-novel-miR-15	DLG1
chi-novel-miR-15	OSBPL11
chi-novel-miR-15	UMPS
chi-novel-miR-15	POPDC2
chi-novel-miR-15	ARHGAP31
chi-novel-miR-15	BTLA
chi-novel-miR-15	PHLDB2
chi-novel-miR-15	KIAA1524
chi-novel-miR-15	TBC1D23
chi-novel-miR-15	CHMP2B
chi-novel-miR-15	MIS18A
chi-novel-miR-15	ATP5O
chi-novel-miR-15	PID1
chi-novel-miR-15	IGFBP6
chi-novel-miR-15	CIDEA
chi-novel-miR-15	RAB18
chi-novel-miR-15	FST
chi-novel-miR-15	HIF1A
chi-novel-miR-15	HSP70.1
chi-novel-miR-15	NPR2
chi-novel-miR-15	SPARC
chi-novel-miR-15	FTH1
chi-novel-miR-141	LRRC15
chi-novel-miR-141	DLG1
chi-novel-miR-141	PARP14
chi-novel-miR-141	GOLGB1
chi-novel-miR-141	RABL3
chi-novel-miR-141	ATP6V1A
chi-novel-miR-141	PHLDB2
chi-novel-miR-141	KIAA1524
chi-novel-miR-141	DCBLD2
chi-novel-miR-141	ZBTB11
chi-novel-miR-141	SENP7
chi-novel-miR-141	TOMM70
chi-novel-miR-141	EPHA3
chi-novel-miR-141	ROBO1
chi-novel-miR-141	HSPA13
chi-novel-miR-141	JAM2

chi-novel-miR-141	USP16
chi-novel-miR-141	MAP3K7CL
chi-novel-miR-141	SLC5A3
chi-novel-miR-141	NFE2L2
chi-novel-miR-141	UCP2
chi-novel-miR-141	AZIN1
chi-novel-miR-141	SP1
chi-novel-miR-141	FABP3
chi-novel-miR-141	NPR2
chi-novel-miR-141	SLC39A1
chi-novel-miR-141	ELOVL5
chi-novel-miR-141	LOC100861279
chi-miR-196b	PEG10
chi-miR-196b	PHLDB2
chi-miR-196b	PLCXD2
chi-miR-196b	KIAA1524
chi-miR-196b	DCBLD2
chi-miR-196b	ROBO1
chi-miR-196b	HSPA13
chi-miR-196b	RWDD2B
chi-miR-196b	SLC5A3
chi-miR-196b	AKR1B1
chi-miR-196b	TNNT3
chi-miR-196b	CTSH
chi-miR-196b	ITGB1
chi-miR-196b	MAN2A1
chi-miR-196b	VEGFA
chi-miR-196b	KANSL2
chi-miR-196b	INSIG1
chi-miR-196b	MMP14
chi-miR-196b	SP1
chi-miR-196b	ASL
chi-miR-196b	P97BCNT
chi-miR-196b	GNS
chi-miR-196b	RHOB
chi-miR-196b	SLC39A1
chi-miR-196b	CAPN1
chi-miR-196b	GHITM
chi-miR-196b	SDF4
chi-miR-146a	PEG10
chi-miR-146a	TBC1D23
chi-miR-146a	EPHA3
chi-miR-146a	CHMP2B

chi-miR-146a	HSPA13
chi-miR-146a	USP16
chi-miR-146a	CCT8
chi-miR-146a	GART
chi-miR-146a	SLC5A3
chi-miR-146a	CTSF
chi-miR-146a	MAN2B1
chi-miR-146a	MEF2A
chi-miR-146a	RBP4
chi-miR-146a	AZIN1
chi-miR-146a	MAN2A1
chi-miR-146a	RHOA
chi-miR-146a	ETS1
chi-miR-146a	PDIA3
chi-miR-146a	HIF1A
chi-miR-146a	GHITM
chi-miR-146a	PRKAG3
chi-miR-146a	OGN
chi-miR-146a	DIMT1
chi-miR-146a	CD36
chi-miR-146a	HMOX1
chi-miR-146a	IL18
chi-miR-128-3p	PEG10
chi-miR-128-3p	CTSF
chi-miR-128-3p	TIMP1
chi-miR-128-3p	IGFBP2
chi-miR-128-3p	MMP9
chi-miR-128-3p	CTSB
chi-miR-128-3p	MEF2A
chi-miR-128-3p	SLC2A4
chi-miR-128-3p	SLC2A1
chi-miR-128-3p	IGFBP3
chi-miR-128-3p	FGF5
chi-miR-128-3p	OAZ2
chi-miR-128-3p	MAN2A1
chi-miR-128-3p	IGF2
chi-miR-128-3p	VEGFA
chi-miR-128-3p	KANSL2
chi-miR-128-3p	RAB7A
chi-miR-128-3p	B4GALT1
chi-miR-128-3p	ETS1
chi-miR-128-3p	FST
chi-miR-128-3p	SP1

chi-miR-128-3p	PNPLA2
chi-miR-128-3p	MANBA
chi-miR-128-3p	GNS
chi-miR-128-3p	RHOB
chi-miR-128-3p	IGFBP4
chi-miR-128-3p	CD9
chi-miR-128-3p	GHITM
chi-miR-128-3p	ELOVL5
chi-let-7a-3p	PEG10
chi-let-7a-3p	XXYLT1
chi-let-7a-3p	PPP1R2
chi-let-7a-3p	POLQ
chi-let-7a-3p	RABL3
chi-let-7a-3p	UPK1B
chi-let-7a-3p	PHLDB2
chi-let-7a-3p	KIAA1524
chi-let-7a-3p	DCBLD2
chi-let-7a-3p	ZBTB11
chi-let-7a-3p	TRMT10C
chi-let-7a-3p	SENP7
chi-let-7a-3p	COL8A1
chi-let-7a-3p	ROBO1
chi-let-7a-3p	MIS18A
chi-let-7a-3p	GART
chi-let-7a-3p	SLC5A3
chi-let-7a-3p	RAB18
chi-let-7a-3p	B4GALT1
chi-let-7a-3p	SDHD
chi-let-7a-3p	HIF1A
chi-let-7a-3p	FABP3
chi-let-7a-3p	P97BCNT
chi-let-7a-3p	MANBA
chi-let-7a-3p	ELOVL5
