# Supplementary material for: Identification of miRNA-mRNA Regulatory Networks Reveals Potential Key miRNAs in Goat Intramuscular and Subcutaneous Adipose Formation
Source: Animals (Basel). 2026 Aug 20;16(16):2603. doi: 10.3390/ani16162603 (PMC13508972; doi:10.3390/ani16162603)
Supplement: Supplementary file 1 [file animals-16-02603-s001.zip › Supplementary Table S4. miRNA–mRNA interaction pairs in the shared regulatory network.pdf]

Supplementary Table S4. miRNA–mRNA interaction pairs in the shared regulatory network.

miRNA	Target mRNA
chi-miR-1	CD36
chi-miR-1	SEPP1
chi-miR-1	PLIN3
chi-miR-1	SCD
chi-miR-1	ELOVL5
chi-miR-1	LOC100860781
chi-miR-1	PPARG
chi-miR-1	SPP1
chi-miR-1	RIDA
chi-miR-1	HIF1A
chi-miR-1	ETS1
chi-miR-1	TMSB4X
chi-miR-1	VEGFA
chi-miR-1	PEG10
chi-miR-1	AZIN1
chi-miR-1	IGFBP3
chi-miR-1	NFE2L2
chi-miR-1	SLC5A3
chi-miR-1	GART
chi-miR-1	SON
chi-miR-1	ATP5J
chi-miR-1	JAM2
chi-miR-1	ROBO2
chi-let-7c-3p	IL18
chi-let-7c-3p	CD36
chi-let-7c-3p	GABARAPL2
chi-let-7c-3p	P97BCNT
chi-let-7c-3p	SDHD
chi-let-7c-3p	SREBF1
chi-let-7c-3p	KANSL2
chi-let-7c-3p	VEGFA
chi-let-7c-3p	BTG3
chi-let-7c-3p	CIDEA
chi-let-7c-3p	NFE2L2
chi-let-7c-3p	SLC5A3
chi-let-7c-3p	SON
chi-let-7c-3p	LTN1
chi-let-7c-3p	HSPA13
chi-let-7c-3p	ROBO2
chi-let-7c-3p	ROBO1

chi-let-7c-3p	CHMP2B
chi-let-7c-3p	EPHA3
chi-let-7c-3p	TOMM70
chi-let-7c-3p	SENP7
chi-let-7c-3p	DCBLD2
chi-let-7c-3p	PPP1R2
chi-miR-129-3p	HMOX1
chi-miR-129-3p	SCD
chi-miR-129-3p	GHITM
chi-miR-129-3p	SLC39A1
chi-miR-129-3p	NPR2
chi-miR-129-3p	CD81
chi-miR-129-3p	GNS
chi-miR-129-3p	ASL
chi-miR-129-3p	SLC2A3
chi-miR-129-3p	MMP14
chi-miR-129-3p	ETS1
chi-miR-129-3p	PEG10
chi-miR-129-3p	AZIN1
chi-miR-129-3p	UCP2
chi-miR-129-3p	ITGB1
chi-miR-129-3p	IGFBP3
chi-miR-129-3p	AKR1B1
chi-miR-129-3p	HSPA2
chi-miR-129-3p	SON
chi-miR-129-3p	MAP3K7CL
chi-miR-129-3p	ROBO1
chi-miR-129-3p	COL8A1
chi-miR-129-3p	PLCXD2
chi-miR-129-3p	PHLDB2
chi-miR-129-3p	ARHGAP31
chi-miR-129-5p	IL18
chi-miR-129-5p	IGFBP5
chi-miR-129-5p	CD36
chi-miR-129-5p	SEPP1
chi-miR-129-5p	DIMT1
chi-miR-129-5p	FASN
chi-miR-129-5p	LOC100860781
chi-miR-129-5p	GNS
chi-miR-129-5p	MANBA
chi-miR-129-5p	P97BCNT
chi-miR-129-5p	FABP3
chi-miR-129-5p	SDHD

chi-miR-129-5p	PDIA3
chi-miR-129-5p	RAB7A
chi-miR-129-5p	TMSB4X
chi-miR-129-5p	VEGFA
chi-miR-129-5p	PEG10
chi-miR-129-5p	ITGB1
chi-miR-129-5p	TNNT2
chi-miR-129-5p	TNNT3
chi-miR-129-5p	SLC2A1
chi-miR-129-5p	HSPA8
chi-miR-129-5p	TNNI3
chi-miR-129-5p	PITX2
chi-miR-133a-3p	INHA
chi-miR-133a-3p	ELOVL5
chi-miR-133a-3p	FASN
chi-miR-133a-3p	RPL21
chi-miR-133a-3p	LOC100860781
chi-miR-133a-3p	PRKAG3
chi-miR-133a-3p	CAPN1
chi-miR-133a-3p	GNS
chi-miR-133a-3p	ASL
chi-miR-133a-3p	RIDA
chi-miR-133a-3p	SREBF1
chi-miR-133a-3p	SP1
chi-miR-133a-3p	MMP14
chi-miR-133a-3p	SLC39A13
chi-miR-133a-3p	KANSL2
chi-miR-133a-3p	CYB5A
chi-miR-133a-3p	OAZ2
chi-miR-133a-3p	SLC2A1
chi-miR-133a-3p	SLC2A4
chi-miR-133a-3p	PITX2
chi-miR-133a-3p	HSPA2
chi-miR-133a-3p	MAN2B1
chi-miR-133a-3p	GART
chi-miR-133a-3p	SON
chi-miR-133a-3p	CCT8
chi-miR-133a-3p	USP16
chi-miR-133a-3p	TIMMDC1
chi-miR-133a-3p	RABL3
chi-miR-133a-5p	DPF2
chi-miR-133a-5p	SLC2A3
chi-miR-133a-5p	TCAP

chi-miR-133a-5p	MMP14
chi-miR-133a-5p	VEGFA
chi-miR-133a-5p	MAN2A1
chi-miR-133a-5p	BTG3
chi-miR-133a-5p	PEG10
chi-miR-133a-5p	AZIN1
chi-miR-133a-5p	CTSH
chi-miR-133a-5p	SLC2A1
chi-miR-133a-5p	MEF2A
chi-miR-133a-5p	TNNI3
chi-miR-133a-5p	CTSB
chi-miR-133a-5p	PITX2
chi-miR-133a-5p	NFE2L2
chi-miR-133a-5p	TIMP1
chi-miR-133a-5p	SLC5A3
chi-miR-133a-5p	SON
chi-miR-133a-5p	USP16
chi-miR-133a-5p	LTN1
chi-miR-133a-5p	HSPA13
chi-miR-133b	INHA
chi-miR-133b	ELOVL5
chi-miR-133b	FASN
chi-miR-133b	RPL21
chi-miR-133b	LOC100860781
chi-miR-133b	PRKAG3
chi-miR-133b	CAPN1
chi-miR-133b	GNS
chi-miR-133b	ASL
chi-miR-133b	RIDA
chi-miR-133b	SREBF1
chi-miR-133b	SP1
chi-miR-133b	SLC39A13
chi-miR-133b	KANSL2
chi-miR-133b	CYB5A
chi-miR-133b	SLC2A1
chi-miR-133b	SLC2A4
chi-miR-133b	PITX2
chi-miR-133b	HSPA2
chi-miR-133b	MAN2B1
chi-miR-133b	COL8A1
chi-miR-133b	PLCXD2
chi-miR-188-5p	FASN
chi-miR-188-5p	NPR2

chi-miR-188-5p	BTG1
chi-miR-188-5p	CHI3L1
chi-miR-188-5p	P97BCNT
chi-miR-188-5p	ASL
chi-miR-188-5p	PDIA3
chi-miR-188-5p	IGF2
chi-miR-188-5p	MAN2A1
chi-miR-188-5p	PEG10
chi-miR-188-5p	CTSH
chi-miR-188-5p	CTSB
chi-miR-188-5p	NFE2L2
chi-miR-188-5p	SON
chi-miR-188-5p	CCT8
chi-miR-188-5p	HSPA13
chi-miR-188-5p	ROBO2
chi-miR-188-5p	EPHA3
chi-miR-188-5p	PROS1
chi-miR-188-5p	COL8A1
chi-miR-188-5p	TBC1D23
chi-miR-188-5p	TOMM70
chi-miR-188-5p	ZBTB11
chi-miR-188-5p	KIAA1524
chi-miR-188-5p	PLCXD2
chi-miR-188-5p	PHLDB2
chi-miR-206	SEPP1
chi-miR-206	PLIN3
chi-miR-206	SCD
chi-miR-206	ELOVL5
chi-miR-206	LOC100860781
chi-miR-206	PPARG
chi-miR-206	SPP1
chi-miR-206	HIF1A
chi-miR-206	ETS1
chi-miR-206	TMSB4X
chi-miR-206	VEGFA
chi-miR-206	AZIN1
chi-miR-206	IGFBP3
chi-miR-206	NFE2L2
chi-miR-206	SLC5A3
chi-miR-206	GART
chi-miR-206	SON
chi-miR-206	ATP5J
chi-miR-206	ROBO2

chi-miR-206	ROBO1
chi-miR-206	TBC1D23
chi-miR-206	ZBTB11
chi-miR-206	KIAA1524
chi-miR-206	PLCXD2
chi-miR-206	PHLDB2
chi-miR-206	SLC35A5
chi-miR-190a-5p	CD36
chi-miR-190a-5p	SEPP1
chi-miR-190a-5p	GHITM
chi-miR-190a-5p	NPR2
chi-miR-190a-5p	SP1
chi-miR-190a-5p	OAZ2
chi-miR-190a-5p	SON
chi-miR-190a-5p	MAP3K7CL
chi-miR-190a-5p	HSPA13
chi-miR-190a-5p	ROBO2
chi-miR-190b	CD200
chi-miR-190b	UPK1B
chi-miR-190b	ARHGAP31
